# Chronic health conditions and school absence, exclusions, and non-enrolment: a cohort study using the Education and Child Health Insights from Linked Data database

**DOI:** 10.1093/pubmed/fdaf064

**Published:** 2025-06-02

**Authors:** Matthew A Jay, Ania Zylbersztejn, Lauren Herlitz, Jessica Deighton, Ruth Gilbert, Ruth Blackburn

**Affiliations:** University College London Great Ormond Street Institute of Child Health, 30 Guilford Street, London WC1N 1EH, UK; University College London Great Ormond Street Institute of Child Health, 30 Guilford Street, London WC1N 1EH, UK; University College London Great Ormond Street Institute of Child Health, 30 Guilford Street, London WC1N 1EH, UK; Social Research Institute, UCL Institute of Education - University College London's Faculty of Education and Society, 55-59 Gordon Square, London WC1H 0NU, UK; The Evidence Based Practice Unit, UCL and Anna Freud, 4-8 Rodney Street, London N1 9JH, UK; University College London Great Ormond Street Institute of Child Health, 30 Guilford Street, London WC1N 1EH, UK; University College London Great Ormond Street Institute of Child Health, 30 Guilford Street, London WC1N 1EH, UK

**Keywords:** epidemiology, education, employment and skills, chronic disease

## Abstract

**Background:**

Policies to reduce school absence can place a burden on children with chronic health conditions (CHCs). Although estimates suggest > 25% of children in England have a CHC before age 16, there is limited evidence on extent of absence, exclusion, and non-enrolment from school among children with CHCs.

**Methods:**

We used all-of-England inpatient data (Hospital Episode Statistics) to identify groups of adolescents with CHCs from age 5 to 15. Cohorts were born in 2000/01 to 2002/03. Data were linked to England’s National Pupil Database for secondary school (age 11 to 16) persistent absence (>1 month missed/year), exclusion, and non-enrolment to examine rates of each outcome by CHC groups.

**Results:**

Of 1 456 361 children, 12.5% had a CHC from age 5 to 11, and 18.9% to age 16. Rates of persistent absence were higher among children with CHCs than unexposed peers (e.g. 25.9% compared to 14.7% aged 15/16), especially among those with mental health presentations (32.1%). Increased rates were found for exclusion and non-enrolment for children with CHCs. The percentage of absence recorded as health-related was lowest among children with externalizing presentations.

**Conclusions:**

Approaches to improve school attendance should consider needs of children with CHCs, ensuring adequate support.

## Introduction

Schools in England have duties to support students with medical conditions, ensuring they remain healthy and safe at school, can fully access education and are not disadvantaged.^1^ Policies to reduce absence, such as attendance targets and fines, have been perceived as punitive and unfair by children with chronic health conditions (CHCs) and their caregivers because health-related absences were unavoidable, they received inadequate support from schools and because other causes of lower attainment were overlooked, e.g. the effect of a CHC or its treatment on cognition, concentration and energy levels, and/or the indirect effects of peer exclusion leading to school disengagement.^2–11^

Over 25% of children in England have a CHC before turning 16,^12^ indicating a need to understand the scale and patterning (e.g. whether predictable, cyclical^4^) of school absence and policy responses. Exploring absence among types of CHC, such as those characterized by physical complaints (e.g. asthma), learning impairments (e.g. neurodisability) or mental health-related presentations, would indicate broadly whether the ‘dose’ of support to engage in schooling may differ depending on condition.

We used all-of-England data to estimate associations between CHCs (reflecting differential functional and cognitive characteristics) and school absence for adolescents attending state schools. We also examined exclusion (permanent and fixed-term) and non-enrolment (de-registration from school), which both cluster in vulnerable groups of children^13–16^ and are important routes through which children miss education. For example, exclusion because of poor conduct and non-enrolment may be indicators that a student’s support needs were unfairly overlooked or could not be met in mainstream schooling.

## Methods

### Data source and population

We used Education and Child Health Insights from Linked Data (ECHILD), which links English health (Hospital Episode Statistics [HES]) and education data (National Pupil Database [NPD]).^17^^,^^18^ HES inpatient records capture clinical and demographic information from all state-funded hospital admissions in England.^19^ Diagnostic and procedure information capturing the main reason for admission and comorbidities is recorded by clinical coders according to national standards (≤20 International Classification of Diseases v10 [ICD-10] codes per episode and ≤ 24 Office of Population Censuses and Surveys v4 [OPCS-4] procedure codes).^20^ NPD includes information on school absences, exclusions, and enrolments for all pupils in English state schools (93% of all pupils each year).^21^

We included all adolescents enrolled in mainstream or special school in year 7 (aged 11/12) in 2012/13, 2013/14, and 2014/15, in any school census (autumn, spring, summer, alternative provision, and pupil referral unit). Children not following the national curriculum (e.g. in special schools) were included if aged 11 in September of the relevant year. The cohorts were born in academic years (September–August) 2000/01 to 2002/03 and reached school leaving age (16) in 2016/17 to 2018/19. We chose these cohorts because absence data are not available for 2019/20 or 2020/21 due to the COVID-19 pandemic. We specified that participants must have linked to 1+ HES records (including outpatients and accident and emergency) because children must be in the country and eligible for NHS services to be included in HES inpatient records. Further, it is possible that there were immigrant adolescents enrolled in year 7 who would not appear in HES before then. As such, we would not be able to detect their CHCs. We therefore specified that participants must have been enrolled at any point from reception, aged 4/5, to year 2, aged 6/7.

### Exposure

We used diagnoses recorded in HES inpatient records^19^ from the reception year (age 4/5) to the end of the academic year preceding the one in which outcomes were measured. For example, for absence in year 7 (age 11/12), CHCs were ascertained from admissions that occurred from reception to the end of year 6 (age 10/11). For absence in year 11 (age 15/16), CHCs were ascertained from reception to the end of year 10 (age 14/15). A code reflects a diagnosis, sign, symptom or underlying condition documented by hospital clinicians as relevant to management during any admission.

We examined adolescents with a CHC generically defined and specific conditions selected for diversity in their potential effects on absence, exclusion, and non-enrolment, whether directly or indirectly, and for their prevalence (Supplementary Table S1). We included physical and mental health presentations under a broad definition of CHCs (Hardelid *et al*.,^22^ which uses 1372 ICD-10 codes), on the basis that there is some commonality of experience among those with CHCs^23^^,^^24^ and to facilitate comparison with other research adopting similar CHC definitions (Supplementary Table S1, group 1).^25^^,^^26^ We separately examined asthma (Supplementary Table S1, 1a), cerebral palsy (1b), diabetes (1c) and epilepsy (1d). Other conditions (severe congenital heart defects, cystic fibrosis, and cleft lip and palate) were unfeasible due to small numbers. We examined neurodisability and associated high-risk conditions (affecting the brain or neuromuscular system creating functional impairment, such as cerebral palsy, epilepsy, intellectual disability) due to increased likelihood of learning disability and other neurodevelopmental problems (Supplementary Table S1, group 2).^27^ We also used broad criteria for identifying mental health-related presentations (group 3), reflecting unplanned hospital admissions indicative of: internalizing (e.g. anxiety, depression; 3a in Supplementary Table S1); externalizing (e.g. substance misuse, disruptive behaviour; 3b); potentially psychosomatic presentations (e.g. breathlessness, fatigue, abdominal pain without a medical cause; 3c)^28^; and adversity (e.g. violence, self-harm, drug or alcohol misuse; 3d^29^). CHCs groups were not mutually exclusive: adolescents could be recorded in multiple CHC subgroups but only once for any CHC overall (Supplementary Table S1, group 0). Machine-readable code lists are available at the ECHILD Phenotype Code List Repository.^30^

### Outcomes

Outcomes were persistent absence, school exclusion, and non-enrolment (Supplementary Table S1). Persistent absence is defined as missing 10% of possible school sessions (~1 month of school per year for a child enrolled full-time).^31^ There are three terms per academic year: autumn (October to December), spring (January to March), and summer (April to July). We used data for all three terms, except for year 11 (age 15/16), where we only used the autumn and spring terms due to higher rates of study leave related to final exams.^32^ Where a child had > 420 possible sessions (210 days), their absence data were set to missing as > 420 possible sessions is unfeasible.^32^ Because adolescents with CHCs are more likely to be ill and attend appointments, we calculated per-child percentages of absence in each year that was recorded by schools as being due to illness or attending healthcare appointments (‘health-related absence’).

Exclusion data is available in yearly NPD modules.^33^ We combined fixed-term and permanent exclusions due to low numbers of the latter. Fixed-term exclusions are temporary suspensions from school (≤45 days per year, most lasting < 5 days^33^) administered in response to breaches of behaviour policy. Permanent exclusions (expulsions) are reserved for serious or persistent breaches and mean that the adolescent leaves the school and must be educated elsewhere. We examined the proportion of adolescents who received any exclusion per year.

Non-enrolment in the spring census of each year was measured across years 8 to 11 (not year 7 as children had to be enrolled then to be included in the study). Non-enrolment in the NPD is a potential marker of off-rolling (illegal exclusion) or otherwise not engaging with education, though it may also be explained by moving to private or home-schooling, emigration or death.^13^ Adolescents were deemed non-enrolled if they were not enrolled in the spring census of each year.

### Demographic variables

In order to describe the cohorts, demographic data were extracted from the NPD on gender, ethnic group, first language, free school meal claims (available due to low parental income) in year 7 and neighbourhood fifth for the income deprivation affecting children index in year 7.^34^

### Analysis

We calculated the cumulative prevalence of each CHC from reception to the end of years 6, 7, 8, 9, and 10 by dividing the number of adolescents with each CHC from reception to each year by the number of adolescents in the cohort. The percentages of adolescents experiencing persistent absence, exclusion, and non-enrolment in each of years 7 (8 in the case of non-enrolment) to 11 were calculated, comparing adolescents with unexposed adolescents (i.e. none of the CHCs studied). We also calculated the percentage of children who experienced each outcome who had a CHC recorded before the relevant year (i.e. the number of children with a CHC experiencing each outcome divided by the total number of children experiencing each outcome). We then calculated the number and percentage of adolescents experiencing none, one or more, one, two, or all three outcomes across years 8 to 11 as a proxy of complexity. Adolescents would be counted, for example, as having three of the outcomes across years 8 to 11 if they experienced each of persistent absence, exclusion, and non-enrolment, though these did not have to be in the same year. Interpretation of this metric should be cautious given that absence and exclusion can only occur when a child is enrolled (meaning that where a child is not enrolled, it is not possible to observe absence or exclusion data).

All analyses were conducted in R. Permissions to use linked, de-identified data from NPD and HES were granted by the Department for Education (DR200604.02B) and NHS England (DARS-NIC-381972). Ethical approval for the ECHILD project was granted by the National Research Ethics Service (17/LO/1494), NHS Health Research Authority Research Ethics Committee (20/EE/0180 and 21/SW/0159) and is overseen by the UCL Great Ormond Street Institute of Child Health’s Joint Research and Development Office (20PE16). Data were accessed through the Office for National Statistics Secure Research Service with statistical disclosure control checks by independent Office for National Statistics analysts.

## Results

There were 1 633 428 adolescents enrolled in year 7, the first year of secondary school, in 2012/13, 2013/14 or 2014/15. Of these, 177 067 (10.8%) were excluded because: they were not born in the relevant years (5558, 0.3%); they were not enrolled in reception to year 2 (80 475, 4.9%); or they did not link to HES (91,034, 5.6%). The analysis cohort consisted of 1 456 361 adolescents (characteristics, Supplementary Table S2).

The cumulative prevalence of any CHC rose from 12.5% to end of year 6 to 18.9% to end of year 10 (Table 1). Mental health-related presentations were the most prevalent subgroup (year 6 to year 10, 3.5% to 7.3%), largely accounted for by psychosomatic presentations (year 6 to year 10, 3.3% to 5.7%).

**Table 1:** Cumulative prevalence of CHCs from school reception (aged 4/5) to the end of years 6 (aged 10/11) to 10 (aged 14/15).

	Cumulative prevalence from start of reception year to end of...				
Condition	Year 6 Aged 10/11	Year 7 Aged 11/12	Year 8 Aged 12/13	Year 9 Aged 13/14	Year 10 Aged 14/15
	*n* (%)	*n* (%)	*n* (%)	*n* (%)	*n* (%)
0. Any CHC	181 832 (12.5%)	201 219 (13.8%)	222 032 (15.2%)	246 746 (16.9%)	275 251 (18.9%)
1. CHC: Hardelid et al	149 004 (10.2%)	163 882 (11.3%)	181 003 (12.4%)	202 328 (13.9%)	227 489 (15.6%)
1a. Asthma	46 479 (3.2%)	51 783 (3.6%)	57 023 (3.9%)	62 569 (4.3%)	68 181 (4.7%)
1b. Cerebral palsy	3724 (0.3%)	3952 (0.3%)	4149 (0.3%)	4367 (0.3%)	4565 (0.3%)
1c. Diabetes	4081 (0.3%)	4755 (0.3%)	5478 (0.4%)	6157 (0.4%)	6789 (0.5%)
1d. Epilepsy	6111 (0.4%)	6651 (0.5%)	7201 (0.5%)	7794 (0.5%)	8442 (0.6%)
2. Neurodisability	27 300 (1.9%)	30 713 (2.1%)	34 166 (2.3%)	38 134 (2.6%)	42 833 (2.9%)
3. Mental health-related	51 686 (3.5%)	61 659 (4.2%)	72 928 (5.0%)	88 108 (6.0%)	106 988 (7.3%)
3a. Internalising	361 (0.0%)	623 (0.0%)	1078 (0.1%)	1796 (0.1%)	3042 (0.2%)
3b. Externalising	228 (0.0%)	375 (0.0%)	769 (0.1%)	1594 (0.1%)	2806 (0.2%)
3c. Psychosomatic	47 545 (3.3%)	56 210 (3.9%)	64 358 (4.4%)	73 279 (5.0%)	83 734 (5.7%)
3d. ARAs	4249 (0.3%)	5729 (0.4%)	9487 (0.7%)	17 357 (1.2%)	28 479 (2.0%)
Unexposed	1 274 529 (87.5%)	1 255 142 (86.2%)	1 234 329 (84.8%)	1 209 615 (83.1%)	1 181 110 (81.1%)
All adolescents	1 456 361 (100.0%)				

ARA, adversity-related admissions; CHC, chronic health condition.

Table 2 shows rates of persistent absence, exclusion, and non-enrolment across secondary school. Rates of persistent absence for all adolescents rose from 10.0% in year 7 to 16.8% in year 11. Those with records of CHCs had a higher risk of persistent absence compared to unaffected peers. For example, in year 11 (final year of secondary school), the percentage of adolescents with any CHC persistently absent was 1.8 times higher than unexposed peers (25.9% vs 14.7%). Adolescents with mental health-related presentations had the highest risk of persistent absence, e.g. 32.1% in year 11. Adolescents with externalizing presentations were at the highest risk of persistent absence (54.0% in year 11) and were also least likely to have absences recorded as health-related (median 45.9% of absence recorded as health-related compared to 86.5% among unexposed peers: Table 3).

**Table 2:** Number and percentage of children experiencing persistent absence, exclusion, and non-enrolment by each chronic health condition.

Condition	Year 7	Year 8	Year 9	Year 10	Year 11
	*n* (%)	*n* (%)	*n* (%)	*n* (%)	*n* (%)
**Persistent absence**					
0. Any CHC	30 077 (16.7%)	38 386 (19.4%)	48 577 (22.5%)	59 044 (24.9%)	67 637 (25.9%)
1. CHC: Hardelid *et al*.	25 530 (17.3%)	32 107 (20.0%)	40 605 (23.1%)	49 617 (25.6%)	57 651 (26.8%)
1a. Asthma	8803 (19.1%)	11 104 (21.8%)	13 647 (24.5%)	15 609 (25.9%)	16 923 (26%)
1b. Cerebral palsy	1065 (29.7%)	1182 (31.6%)	1275 (33.0%)	1423 (35.2%)	1459 (35.2%)
1c. Diabetes	1022 (25.1%)	1293 (27.6%)	1572 (29.4%)	1812 (30.4%)	1967 (30.2%)
1d. Epilepsy	1579 (26.7%)	1755 (27.6%)	2027 (29.8%)	2323 (32.0%)	2512 (32.5%)
2. Neurodisability	6387 (24.3%)	7672 (26.4%)	9147 (28.7%)	10 694 (30.5%)	12 391 (32.2%)
3. Mental health-related	9291 (18.1%)	13 167 (21.8%)	18 427 (26.1%)	25 323 (30.2%)	32 247 (32.1%)
3a. Internalising	96 (27.0%)	190 (32.5%)	395 (39.8%)	713 (44.0%)	1257 (45.9%)
3b. Externalising	73 (35.4%)	143 (43.7%)	363 (54.6%)	781 (56.7%)	1306 (54.0%)
3c. Psychosomatic	8435 (17.8%)	11 697 (21.1%)	15 247 (24.3%)	18 941 (26.9%)	22 315 (28.1%)
3d. ARAs	924 (22.4%)	1648 (30.3%)	3764 (42.7%)	7809 (49.6%)	12 850 (50.7%)
Unexposed	114 765 (9%)	135 690 (10.9%)	160 306 (13.2%)	172 993 (14.7%)	168 467 (14.7%)
All adolescents	144 842 (10%)	174 076 (12.1%)	208 883 (14.6%)	232 037 (16.4%)	236 104 (16.8%)
**Exclusion**					
0. Any CHC	6116 (3.4%)	10 681 (5.3%)	16 115 (7.3%)	21 297 (8.6%)	18 507 (6.7%)
1. CHC: Hardelid *et al*.	5027 (3.4%)	8667 (5.3%)	13 132 (7.3%)	17 486 (8.6%)	15 337 (6.7%)
1a. Asthma	1770 (3.8%)	3041 (5.9%)	4477 (7.9%)	5660 (9.0%)	4769 (7.0%)
1b. Cerebral palsy	46 (1.2%)	65 (1.6%)	79 (1.9%)	115 (2.6%)	87 (1.9%)
1c. Diabetes	86 (2.1%)	169 (3.6%)	278 (5.1%)	375 (6.1%)	316 (4.7%)
1d. Epilepsy	160 (2.6%)	248 (3.7%)	347 (4.8%)	427 (5.5%)	317 (3.8%)
2. Neurodisability	1213 (4.4%)	1844 (6.0%)	2529 (7.4%)	3081 (8.1%)	2683 (6.3%)
3. Mental health-related	1814 (3.5%)	3607 (5.8%)	6069 (8.3%)	9059 (10.3%)	8508 (8.0%)
3a. Internalizing	13 (3.6%)	36 (5.8%)	77 (7.1%)	122 (6.8%)	156 (5.1%)
3b. Externalizing	50 (21.9%)	93 (24.8%)	241 (31.3%)	443 (27.8%)	510 (18.2%)
3c. Psychosomatic	1419 (3.0%)	2784 (5.0%)	4428 (6.9%)	6062 (8.3%)	5290 (6.3%)
3d. ARAs	408 (9.6%)	890 (15.5%)	1893 (20%)	3585 (20.7%)	3968 (13.9%)
Unexposed	33 906 (2.7%)	54 089 (4.3%)	72 685 (5.9%)	83 226 (6.9%)	62 778 (5.3%)
All adolescents	40 022 (2.7%)	64 770 (4.4%)	88 800 (6.1%)	104 523 (7.2%)	81 285 (5.6%)
**Non-enrolment, spring census** ^a^					
0. Any CHC	–	2119 (1.1%)	4034 (1.8%)	7150 (2.9%)	12 596 (4.6%)
1. CHC: Hardelid *et al*.	–	1739 (1.1%)	3308 (1.8%)	5921 (2.9%)	10 575 (4.6%)
1a. Asthma	–	540 (1.0%)	1025 (1.8%)	1720 (2.7%)	2864 (4.2%)
1b. Cerebral palsy	–	71 (1.8%)	118 (2.8%)	182 (4.2%)	230 (5.0%)
1c. Diabetes	–	43 (0.9%)	105 (1.9%)	176 (2.9%)	290 (4.3%)
1d. Epilepsy	–	92 (1.4%)	174 (2.4%)	285 (3.7%)	421 (5.0%)
2. Neurodisability	–	417 (1.4%)	758 (2.2%)	1295 (3.4%)	2222 (5.2%)
3. Mental health-related	–	769 (1.2%)	1605 (2.2%)	3144 (3.6%)	6322 (5.9%)
3a. Internalizing	–	16 (2.6%)	46 (4.3%)	106 (5.9%)	292 (9.6%)
3b. Externalizing	–	17 (4.5%)	44 (5.7%)	129 (8.1%)	325 (11.6%)
3c. Psychosomatic	–	639 (1.1%)	1300 (2.0%)	2295 (3.1%)	4118 (4.9%)
3d. ARAs	–	131 (2.3%)	358 (3.8%)	1067 (6.1%)	2886 (10.1%)
Unexposed	–	10 886 (0.9%)	19 313 (1.6%)	27 943 (2.3%)	38 812 (3.3%)
All adolescents	–	13 005 (0.9%)	23 347 (1.6%)	35 093 (2.4%)	51 408 (3.5%)

ARA adversity-related admissions; CHC chronic health condition.

a Non-enrolment data for year 7 are not presented as adolescents had to be enrolled in year 7 to be included in the study.

**Table 3:** Per-child percentage of absence that was due to illness or attending healthcare appointments by each chronic health condition group.

Condition	Year 7	Year 8	Year 9	Year 10	Year 11
	Median (IQR)	Median (IQR)	Median (IQR)	Median (IQR)	Median (IQR)
0. Any CHC	91.8 (63.3, 100)	88.4 (57.4, 100)	85.1 (51.8, 100)	80.6 (45.5, 100)	83.6 (44.3, 100)
1. CHC: Hardelid et al	92.1 (63.8, 100)	88.7 (57.9, 100)	85.3 (52.1, 100)	80.8 (45.5, 100)	83.5 (44.1, 100)
1a. Asthma	90.7 (63.7, 100)	87.2 (56.3, 100)	84.1 (50.8, 100)	79.6 (45.5, 100)	83.3 (44.2, 100)
1b. Cerebral palsy	96.8 (66.7, 100)	96.0 (66.3, 100)	91.4 (63.3, 100)	87.5 (59.0, 100)	91.1 (64.3, 100)
1c. Diabetes	97.0 (77.6, 100)	94.4 (72.7, 100)	92.6 (68.6, 100)	90.1 (62.5, 100)	92.9 (62.8, 100)
1d. Epilepsy	95.9 (61.6, 100)	93.0 (56.8, 100)	88.4 (56.8, 100)	84.2 (51.7, 100)	88.5 (54.5, 100)
2. Neurodisability	92.5 (59.7, 100)	89.3 (51.8, 100)	85.3 (50.0, 100)	80.8 (44.8, 100)	83.3 (44.8, 100)
3. Mental health-related	91.0 (62.5, 100)	87.5 (56.5, 100)	83.3 (50.0, 100)	77.7 (42.2, 100)	80.0 (38.3, 100)
3a. Internalizing	91.5 (61.5, 100)	86.8 (52.5, 100)	80.7 (45.0, 100)	76.2 (40.5, 97.7)	76.2 (34.0, 100)
3b. Externalizing	69.0 (29.6, 92.0)	50.9 (17.5, 86.0)	41.5 (10.3, 78.7)	40.1 (10.2, 76.5)	45.9 (9.5, 87.1)
3c. Psychosomatic	91.6 (64.5, 100)	88.4 (59.3, 100)	85.3 (53.5, 100)	81.0 (46.8, 100)	84.3 (46.7, 100)
3d. ARAs	80.0 (45.3, 100)	70.4 (31.8, 100)	61.8 (24.6, 92.1)	54.7 (19.6, 87.5)	56.0 (17.0, 91.0)
Unexposed	92.2 (61.5, 100)	89.1 (55.8, 100)	86.2 (51.4, 100)	81.7 (45.5, 100)	86.5 (46.7, 100)
All adolescents	92.2 (61.5, 100)	88.9 (56.1, 100)	86.0 (51.5, 100)	81.4 (45.5, 100)	85.9 (46.5, 100)

ARA, adversity-related admissions; CHC, chronic health condition.

Across all groups, the risk of exclusion rose across secondary school, peaking in year 10 (Table 2). Across years 7 to 11, the risk of exclusion among adolescents with any CHC was 1.2 to 1.3 times higher than among unexposed adolescents. Rates were highest among those with externalizing presentations (21.9% in year 7, 27.8% in year 10, and 18.2% in year 11; 8.1, 4.0, 3.4 times higher than unexposed peers). Exclusion rates were lower across secondary school among adolescents with records of cerebral palsy, diabetes or epilepsy.

The risk of non-enrolment increased among all groups from year 8 to 11 (Table 2). Between 0.9% (year 8) and 3.5% (year 11) of all adolescents experienced non-enrolment, compared to 1.1% to 4.6% of adolescents with any CHC. Adolescents with mental health presentations were most at risk, especially those with internalizing or externalizing presentations or adversity-related admissions (e.g. 9.6%, 11.6%, and 10.1% in year 11, respectively).

Among all adolescents, 34.5% experienced at least one of the outcomes of persistent absence, exclusion, or non-enrolment: 23.6% had one, 9.6% had two, and 1.3% had all three (Table 4). The percentages of adolescents with multiple outcomes were higher among all CHC groups and highest among those with externalizing presentations, where 78.6% had one or more outcomes.

**Table 4:** The number and percentage of children in each chronic health condition group experience none, one, two, or all three of persistent absence, exclusion, and/or non-enrolment across years 8 to 11.

Condition	Number of outcomes (persistent absence, exclusion, and/or non-enrolment)				
	0	1 or more	1	2	3
	n (%)	n (%)	n (%)	n (%)	n (%)
0. Any CHC	98 745 (55.8%)	78 210 (44.2%)	54 883 (31.0%)	20 840 (11.8%)	2487 (1.4%)
1. CHC: Hardelid et al	80 030 (55.3%)	64 792 (44.7%)	45 838 (31.7%)	16 967 (11.7%)	1987 (1.4%)
1a. Asthma	23 953 (52.7%)	21 511 (47.3%)	14 587 (32.1%)	6176 (13.6%)	748 (1.6%)
1b. Cerebral palsy	1390 (39.9%)	2094 (60.1%)	1835 (52.7%)	248 (7.1%)	11 (0.3%)
1c. Diabetes	1982 (49.4%)	2027 (50.6%)	1553 (38.7%)	436 (10.9%)	38 (0.9%)
1d. Epilepsy	2558 (44.4%)	3200 (55.6%)	2590 (45.0%)	564 (9.8%)	46 (0.8%)
2. Neurodisability	11 698 (45.8%)	13 820 (54.2%)	10 602 (41.5%)	2925 (11.5%)	293 (1.1%)
3. Mental health-related	27 040 (53.7%)	23 298 (46.3%)	16 096 (32.0%)	6393 (12.7%)	809 (1.6%)
3a. Internalizing	166 (47.8%)	181 (52.2%)	117 (33.7%)	^a^	^a^
3b. Externalizing	42 (21.4%)	154 (78.6%)	76 (38.8%)	63 (32.1%)	15 (7.7%)
3c. Psychosomatic	25 346 (54.6%)	21,076 (45.4%)	14 920 (32.1%)	5485 (11.8%)	671 (1.4%)
3d. ARAs	1679 (41.7%)	2345 (58.3%)	1250 (31.1%)	953 (23.7%)	142 (3.5%)
Unexposed	835 793 (66.9%)	413 506 (33.1%)	281 504 (22.5%)	116 319 (9.3%)	15 683 (1.3%)
All adolescents	934 538 (65.5%)	491 716 (34.5%)	336 387 (23.6%)	137 159 (9.6%)	18 170 (1.3%)

ARA, adversity-related admissions; CHC, chronic health condition.

a Suppressed due to small cell count.

Supplementary Fig. S1 shows the percentage of children experiencing each outcome who had a CHC recorded before each year, showing that 20.8% of children experiencing persistent absence in year 7 had a CHC recorded in primary school. This rose to 28.6% in year 11. For exclusion, these figures were 15.3% in year 7, to 22.8% in year 11, and for non-enrolment, 16.3% in year 8 to 24.5% in year 11.

## Discussion

### Main finding of this study

Adolescents with CHCs recorded in hospital data were at higher risk of persistent absence, exclusion, and non-enrolment than peers without, indicating that needs were not met by schools and that absence and exclusion are potentially modifiable mechanisms through which achievement gaps might be prevented. Depending on condition, around 25% to 50% of adolescents with CHCs were persistently absent in the final two school years aged 14–16, compared to 15% of those without. Rates of persistent absence, exclusion, and non-enrolment were highest among adolescents with mental health-related conditions, suggesting it was difficult for schools to support these students.

### What is already known on this topic

School absence has remained high since the COVID-19 pandemic despite policies, such as attendance targets and fines, to reduce it.^31^^,^^35–37^ There are concerns about how such policies may disadvantage children with CHCs with qualitative work showing how school policy can help or hinder such children.^2–10^ Our findings our similar to data from New South Wales showing children with CHCs (per Hardelid et al.^22^) were more likely to miss testing or underperform on tests, which implies needs were not being met.^38^ Results are also similar to a Welsh primary and secondary care data study, which found similar rates of persistent absence (25% to 50%) and similar rates of exclusion (5% to over 12.5%), depending on secondary school year and condition.^39^

### What this study adds

Findings raise questions concerning equity and justice in the provision of education in England. Children with CHCs are disadvantaged compared to peers in being substantially more likely to miss education.^2^^,^^3^^,^^5^ Schools and the state more broadly have legal duties to ensure the right to education of all children (a fundamental human right^40^) but our findings indicate that this is not being achieved equitably for all groups.

Policy to improve educational participation should address differing needs of children with CHCs, including helping adolescents with mental health-related conditions to thrive. Possible forms of support for adolescents were explored in a qualitative study by Herlitz *et al*.,^11^ showing that, while examples of good practice exist, accessing support can be difficult. Adolescents in our study were recorded as having CHCs if they had admissions with a relevant diagnosis prior to the academic year in question meaning they were known to healthcare and could have been known to schools, e.g. through joined-up working. This in turn could lead to better identification and responses to the needs of such children. Naturally, this requires adequate resourcing of both healthcare and education services. Systematic factors should also be examined, such as school inspection mechanisms based around attendance and exam results and the ways in which these result in pressure on students, in turn affecting their health, well-being, and ability to engage in education.^2–10^

Persistent absence rates for adolescents with CHCs were high and rose as school continued. Particularly for mental health-related presentations, persistent absence rates rose sharply between years 7 and 8, and again between years 8 and 9, suggesting that schools should engage families early when problems arise to understand their perspectives on the causes for absence and address modifiable factors impacting on school engagement, e.g. bullying and peer exclusion, and falling behind with learning as a result of absence.^3^^,^^4^^,^^24^ Non-enrolment was more frequent across all groups, especially among adolescents with mental health-related presentations. Mechanisms for non-enrolment are unclear but it occurs more frequently in other vulnerable groups, including those with social care involvement, special educational needs or living with income deprivation.^13^ Maintaining a register of children not in school, as announced in the 2024 King’s Speech,^41^ could enable monitoring and research on outcomes for such pupils if linkable to the NPD, which would be further enhanced if data on pupils in private schools were available.

### Limitations of this study

While we used generic definitions of CHCs, widening applicability of results to services and policy,^23^ using inpatient data will have led to under-ascertainment of some CHCs, particularly mental health and those seen only in the community, outpatients or emergency departments. There may also have been exposure misclassification, such that adolescents were admitted to hospital but did not have a CHC recorded. This was mitigated by our inclusion of internalizing, externalizing, and psychosomatic presentations and adversity-related admissions, which would capture problems not otherwise diagnosed or not recognized by acute services without specialist input. However, these, too, only capture a sharp end of severity as they exclude such presentations to primary care or the emergency department. We were unable to examine the severity or duration of CHCs. Although we adopted a landmark approach whereby exposure ascertainment was updated relative to the timing of outcomes, we could not explore their relationship between disease onset and recovery. However, the study is generalizable to state schools in England as we used national data with linkage rates of 95%.^18^ Data quality was high as diagnostic, absence, exclusions, and enrolment data are mandatory returns, collected under national standards, undergoing routine validation, and used for planning and remuneration.^21^^,^^42^ CHCs and educational outcomes are socially patterned but it was beyond the scope to investigate how intersecting sociodemographic factors, such as deprivation^43^^,^^44^ and ethnicity,^45^^,^^46^ might affect educational outcomes among children with CHCs. Cohort and qualitative studies are needed to understand the extent and reasons for intersecting health and education inequalities.

## Supplementary Material

Supplementary_Material_fdaf064_amended

## Data Availability

This study uses the ECHILD database, which includes data from the Hospital Episode Statistics and National Pupil Database. ECHILD is open to access by researchers at UK institutions via https://echild.ac.uk. The phenotype code lists used in this study are available at https://code.echild.ac.uk.

## Notes

**Matthew A. Jay**, Senior Research Fellow

**Ania Zylbersztejn**, Senior Research Fellow

**Lauren Herlitz**, Research Fellow

**Jessica Deighton**, Professor of Child Mental Health and Wellbeing

**Ruth Gilbert**, Professor of Clinical Epidemiology

**Ruth Blackburn**, Senior Research Fellow
