# Supplementary material for: Chronic health conditions and school absence, exclusions, and non-enrolment: a cohort study using the Education and Child Health Insights from Linked Data database
Source: J Public Health (Oxf). 2025 Jun 2;47(3):414–22. doi: 10.1093/pubmed/fdaf064 (PMC12395948; doi:10.1093/pubmed/fdaf064)
Supplement: Supplementary_Material_fdaf064_amended [file supplementary_material_fdaf064_amended.docx]

Table S1. Chronic health condition phenotypes and outcomes used in the study

| **Condition** | **Definition** |
| --- | --- |
| 0. Any CHC | Any of groups 1 to 3, below (children with more than one CHC were only counted once in this group, but counted each time recorded in sub-group analyses) |
|  |  |
| 1. CHCs (Hardelid et al^1^) | Any health problem likely to require follow-up for more than 1 year recorded in HES inpatients (n=1372 ICD-10 codes): Hardelid et al.^1^ Sub-groups 1a to 1d are four of the specific conditions, not all conditions in this phenotype. Available at <https://code.echild.ac.uk/chc_hardelid_v1>. |
|  |  |
| *1a. Asthma* | *Asthma codes (n=2) as used by Lut et al^2^ (these codes also appear in Hardelid et al,^1^ but Lut et al^2^ apply codes to exclude non-asthmatic respiratory conditions). Available at* [*https://code.echild.ac.uk/asthma_lut_v1*](https://code.echild.ac.uk/asthma_lut_v1)*.* |
|  | . |
| *1b. Cerebral palsy* | *Cerebral palsy codes (n=7, Hardelid et al.^1^)* |
|  |  |
| *1c. Diabetes* | *Diabetes codes (n=12, Hardelid et al.^1^)* |
|  |  |
| *1d. Epilepsy* | *Epilepsy codes (n=18, Hardelid et al.^1^)* |
|  |  |
| 2. Neurodisability | Neurodisability and associated high risk conditions, i.e., conditions affecting the brain or neuromuscular system creating functional impairment, such as cerebral palsy, epilepsy, intellectual disability, autism, sensory impairment and conditions affecting motor function (n=638 ICD-10 and 14 OPCS-4 codes): Zylbersztejn et al.^3^ Available at <https://github.com/UCL-ECHILD/phenotype-code-lists/blob/main/neurodis_zylb_v1.csv> |
|  |  |
| 3. Mental health-related presentations | Any of 3a to 3d (all recorded in unplanned admissions only). |
|  |  |
| *3a. Internalising presentations* | *Internalising presentations such as anxiety and depression (n=28 ICD-10 codes): Ní Chobhthaigh et al.^4^ Available at* [*https://code.echild.ac.uk/srp_nichobhthaigh_v2*](https://code.echild.ac.uk/srp_nichobhthaigh_v2)*.* |
|  |  |
| *3b. Externalising presentations* | *Externalising presentations such as conduct disorders and drug and alcohol use (n=42 ICD-10 codes): Ní Chobhthaigh et al.^4^ We included in this group a small number of children with conditions categorised by Ní Chobhthaigh et al as thought disorders as numbers were too small to report results separately. This did not change results for the externalising group overall.* |
|  |  |
| *3c. Potentially psychosomatic presentations* | *Potentially psychosomatic presentations such as medically unexplained pain and sleep disorders (n=50 ICD-10 codes): Ní Chobhthaigh et al.^4^* |
|  |  |
| *3d. Adversity-related admissions (ARA)* | *Adversity-related admissions: violence, self-harm or drug or alcohol misuse (n=152 ICD-10 codes): Herbert et al.^5^ Available at* [*https://code.echild.ac.uk/ari_herbert_v1*](https://code.echild.ac.uk/ari_herbert_v1)*. We did not require injuries as in Herbert et al.^5^* |
|  |  |
| **Outcome** | **Definition** |
| Persistent absence | Missing 10% or more of possible school sessions in an academic year, officially recorded. This equates to about a month of school for a child enrolled full-time.^6^ |
|  |  |
| Exclusion | Either a fixed-term (temporary) or permanent exclusion from school, administered in response to breach of school discipline and officially recorded.^7^ |
|  |  |
| Non-enrolment | Becoming un-enrolled from the spring census. This could be due to off-rolling (illegal and unrecorded exclusion), disengaging from education, transfer to home or private school, emigration or death.^8^ It is not possible to calculate the duration of schooling missed as a result of non-enrolment. |
|  |  |

ARA adversity-related admissions; CHC chronic health condition; ICD-10 International Statistical Classification of Diseases and Related Health Problems, version 10 (diagnosis codes); OPCS-4 Office for Population Censuses and Surveys Classification of Interventions and Procedures, version 4 (procedure codes).

Table S2. Cohort demographics

|  | **Inception year (year 7, age 11/12)** | | | **Total** |
| --- | --- | --- | --- | --- |
| **Characteristic** | 2013, N = 478,155 | 2014, N = 476,456 | 2015, N = 501,750 | N = 1,456,361 |
|  | n (%) | n (%) | n (%) | n (%) |
|  |  |  |  |  |
| Female | 231,922 (49%) | 229,931 (48%) | 243,239 (48%) | 705,092 (48%) |
| Unknown | 24 | 29 | 40 | 93 |
|  |  |  |  |  |
| Ethnicity |  |  |  |  |
| White | 385,364 (81%) | 378,607 (80%) | 394,535 (79%) | 1,158,506 (80%) |
| Black | 22,169 (4.6%) | 22,939 (4.8%) | 25,903 (5.2%) | 71,011 (4.9%) |
| Mixed | 19,947 (4.2%) | 21,176 (4.4%) | 23,494 (4.7%) | 64,617 (4.4%) |
| Asian | 42,408 (8.9%) | 45,260 (9.5%) | 48,342 (9.6%) | 136,010 (9.3%) |
| Other | 7,717 (1.6%) | 7,901 (1.7%) | 8,895 (1.8%) | 24,513 (1.7%) |
| Unknown | 550 | 573 | 581 | 1,704 |
|  |  |  |  |  |
| First language |  |  |  |  |
| English | 411,316 (86%) | 405,014 (85%) | 423,763 (84%) | 1,240,093 (85%) |
| Not English | 66,804 (14%) | 71,384 (15%) | 77,930 (16%) | 216,118 (15%) |
| Unknown | 35 | 58 | 57 | 150 |
|  |  |  |  |  |
| Free school meals year 7 (age 11/12) | 97,965 (20%) | 93,980 (20%) | 92,202 (18%) | 284,147 (20%) |
|  |  |  |  |  |
| IDACI fifth year 7 (age 11/12) |  |  |  |  |
| 5 | 114,412 (24%) | 115,457 (24%) | 123,125 (25%) | 352,994 (24%) |
| 4 | 96,448 (20%) | 96,956 (20%) | 101,366 (20%) | 294,770 (20%) |
| 3 | 90,236 (19%) | 88,885 (19%) | 94,257 (19%) | 273,378 (19%) |
| 2 | 89,744 (19%) | 88,754 (19%) | 92,707 (19%) | 271,205 (19%) |
| 1 | 86,042 (18%) | 84,964 (18%) | 88,951 (18%) | 259,957 (18%) |
| Unknown | 1,273 | 1,440 | 1,344 | 4,057 |
|  |  |  |  |  |

Free school meals are available to children in families with low income. IDACI Income domain affecting children index, a neighbourhood-level metric of financial deprivation.

Figure S1. Percentage of children experiencing each outcome with any chronic health condition recorded prior to each year


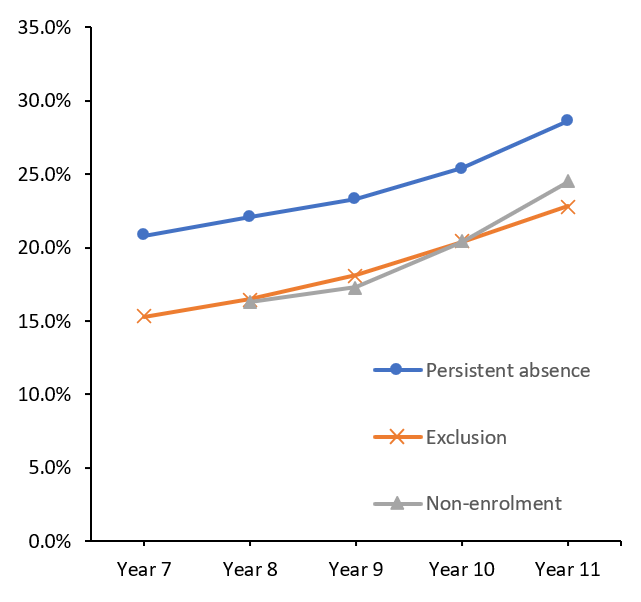


**References**

1. Hardelid P, Dattani N, Gilbert R. Estimating the prevalence of chronic conditions in children who die in England, Scotland and Wales: a data linkage cohort study. *BMJ Open*. 2014;4(8):e005331.

2. Lut I, Lewis K, Wijlaars L, Gilbert R, Fitzpatrick T, Lu H, et al. Challenges of using asthma admission rates as a measure of primary care quality in children: An international comparison. *Journal of Health Services Research & Policy*. 2021;26(4):251-262.

3. Zylbersztejn A, Nguyen V, Gilbert R, Harron K. P102 Special educational needs of primary school aged children with neurodevelopmental conditions: a population cohort study using linked health and education records. *Journal of Epidemiology and Community Health*. 2023;77(Suppl 1):A100.

4. Ní Chobhthaigh S, Jay M, Blackburn R. Emergency hospital admissions for stress-related presentations among secondary school-aged minoritised young people in England. *Br J Psych (in press)*. 2024.

5. Herbert A, Gilbert R, González-Izquierdo A, Li L. Violence, self-harm and drug or alcohol misuse in adolescents admitted to hospitals in England for injury: a retrospective cohort study. *BMJ Open*. 2015;5(2):e006079.

6. Department for Education. Statistics: pupil attendance and absence. <https://www.gov.uk/government/collections/statistics-pupil-absence>. Published 2024. Accessed 25 June 2024.

7. Department for Education. Suspensions and permanent exclusions in England. <https://explore-education-statistics.service.gov.uk/find-statistics/suspensions-and-permanent-exclusions-in-england>. Published 2024. Accessed 25 June 2024.

8. Jay MA, Grath-Lone LM, De Stavola B, Gilbert R. Evaluation of pushing out of children from all English state schools: Administrative data cohort study of children receiving social care and their peers. *Child Abuse & Neglect*. 2022;127:105582.
